# Experience of using adalimumab in treating sight-threatening paediatric or adolescent Behcet’s disease-related uveitis

**DOI:** 10.1186/s12348-019-0181-z

**Published:** 2019-07-31

**Authors:** Mary Ho, Li Jia Chen, Helena P. Y. Sin, Lawrence P. L. Iu, Marten Brelen, Assunta C. H. Ho, Timothy Y. Y. Lai, Alvin L. Young

**Affiliations:** 1 Department of Ophthalmology & Visual Sciences, Prince of Wales Hospital, the Chinese University of Hong Kong, Shatin, Hong Kong SAR; 2 Department of Paediatrics, Faculty of Medicine, the Chinese University of Hong Kong, Shatin, Hong Kong SAR; 3 Department of Ophthalmology & Visual Sciences, Hong Kong Eye Hospital, the Chinese University of Hong Kong, Shatin, Hong Kong SAR

**Keywords:** Behcet’s disease, Adalimumab, Intraocular inflammation, Retinal vasculitis, Uveitis

## Abstract

**Purpose:**

To report the clinical outcomes of adalimumab in treating refractory Behcet’s disease (BD)-related uveitis in paediatric or adolescent patients.

**Methods:**

Retrospective review of five paediatric or adolescent patients with BD-related uveitis with a minimum follow-up of 24 months.

**Results:**

Disease quiescence was observed in 9 (90%) of 10 eyes at 12 months. The mean number of relapses per year per patient was 5 (range, 3–7) before initiation of adalimumab treatment. This was reduced to 0.2 relapse per patient per year among the five patients during the first 24 months after starting adalimumab treatment. At baseline, 5 eyes had active retinal vasculitis. Retinal vasculitis resolved in all cases (100%) after starting adalimumab. The mean time to complete resolution of inflammation was 3.4 weeks. The mean ± standard deviation logMAR best-corrected visual acuity was 0.711 ± 0.63 at baseline and improved to 0.172 ± 1.04 at 12 months (*P* < 0.001). None of the patients developed any adverse events associated with adalimumab treatment.

**Conclusion:**

Adalimumab was effective in preventing irreversible sight-threatening BD-related uveitis in paediatric or adolescent patients. Adalimumab appears to be a promising treatment option for young patients with recalcitrant BD-related uveitis and has a favourable safety profile.

## Introduction

Behcet’s disease (BD) is a multisystem inflammatory disorder with vasculitic changes affecting different parts of the body [9]. The ocular features include posterior uveitis, panuveitis and even occlusive retinal vasculitis [21]. The incidence of uveitis in Behcet’s disease was reported to be 30% in children and 60–80% in adults [16, 37, 42, 43]. Its clinical course in paediatric age group is more aggressive than in adults, characterized by recurrent severe episodes and high rates of permanent visual loss [38].

Treatment with high-dose corticosteroids, with concomitant use of immunosuppressant, is generally able to result in prompt remission of BD-related uveitis. However, the use of corticosteroid has to be judicious in children because of its significant side effects including steroid dependence, Cushing syndrome and impaired bone growth [9, 33]. Despite aggressive therapy, blindness occurs in 16–25% of patients after 5 years of disease onset [37, 38]. Moreover, a subgroup of patients can develop sight-threatening complications, as well as intolerance to standard immunosuppressive agents. In these cases, tumour necrotic factor (TNF) alpha blockade emerges as a valid therapeutic option, as high levels of TNF alpha and its soluble receptor have been detected in the serum and aqueous humour of patients with active BD [10, 39]. Infliximab and adalimumab, both anti-TNF alpha antibodies, have been reported to successfully control BD-related uveitis [24, 35]. Data from systematic review showed that remission rate with anti-TNF alpha agents use is as high as 60–100% in 369 adult patients with BD-related uveitis [3]. These data leads to expert panel recommendations on anti-TNF alpha agents as the first or second-line treatment for BD-related ophthalmic symptoms in adults [18, 30].

For paediatric patients, adalimumab has been evaluated in juvenile idiopathic arthritis (JIA)-associated uveitis, [8, 28, 29], as well as non-infectious uveitis in general [5–7, 11, 14, 23, 31, 40, 41]. The purpose of our study is to report the clinical outcome of adalimumab in a small case series of sight-threatening paediatric or adolescent onset BD-related uveitis.

## Patient and Methods

A retrospective review of records of patients with BD treated with adalimumab between June 2012 and June 2018 was carried out in the Prince of Wales Hospital Eye Center. Informed consent was obtained from patients and parents for publication. The patients were diagnosed based on the International Study Group criteria for BD. All patients in the current series were started on anti-TNF alpha agents when they developed sight-threatening relapse episodes once oral prednisolone was reduced to less than 15 mg while on at least 2 other immunosuppressants of maximum dose. Sight-threatening relapse included focal or multi-focal retinitis or evidence of retinal vasculitis.

The risks and benefits of adalimumab and alternative therapies were fully explained, and consent for treatment was obtained from the parents of patients. Adalimumab was administered to all patients by subcutaneous injections of 40 mg every 2 weeks. Adalimumab was prescribed as a self-financed item and all medications were either self-funded by patients or funded by charity organization. All patients had a complete physical examination, blood tests on complete blood count, renal and liver function test, hepatitis B/C virus status, ANA level, urine analysis and tuberculosis work up including chest x-ray and quantiferon blood test. Patients’ general health, growth, medication toxicity, steroid-related side effects and adverse events related to adalimumab were documented by paediatric rheumatologists on a regular basis.

All patients underwent a complete ophthalmologic examination and were managed by the same team of uveitis clinicians monthly. Severity of ocular involvement and response to treatment were evaluated according to the Standardization of Uveitis Nomenclature (SUN) Workgroup criteria [13]. Complete response was defined as presence of < 0.5+ cellular reaction in the anterior chamber and vitreous humour. Retinal vasculitis was evaluated with a score from 0 to 3 on fundus examination and fluorescein angiography (0 = absence of vasculitis, 1 = vasculitis of peripheral retinal vessels, 2 = posterior pole vasculitis, 3 = vasculitis with evidence of white patches of retinitis). Ocular relapse of BD was defined as at least 50% increase in inflammation and retinal vasculitis scores. Complete control was defined as complete disease quiescence without any signs of ocular inflammation for at least 3 months. Fluorescein angiograms were performed if clinicians suspect an ocular relapse of BD.

The primary outcome was defined as the number of relapses after adalimumab; secondary outcomes were the time to control of inflammatory activity, best-corrected visual acuity and severity of inflammation in each relapse.

## Results

**Fig. 1:**
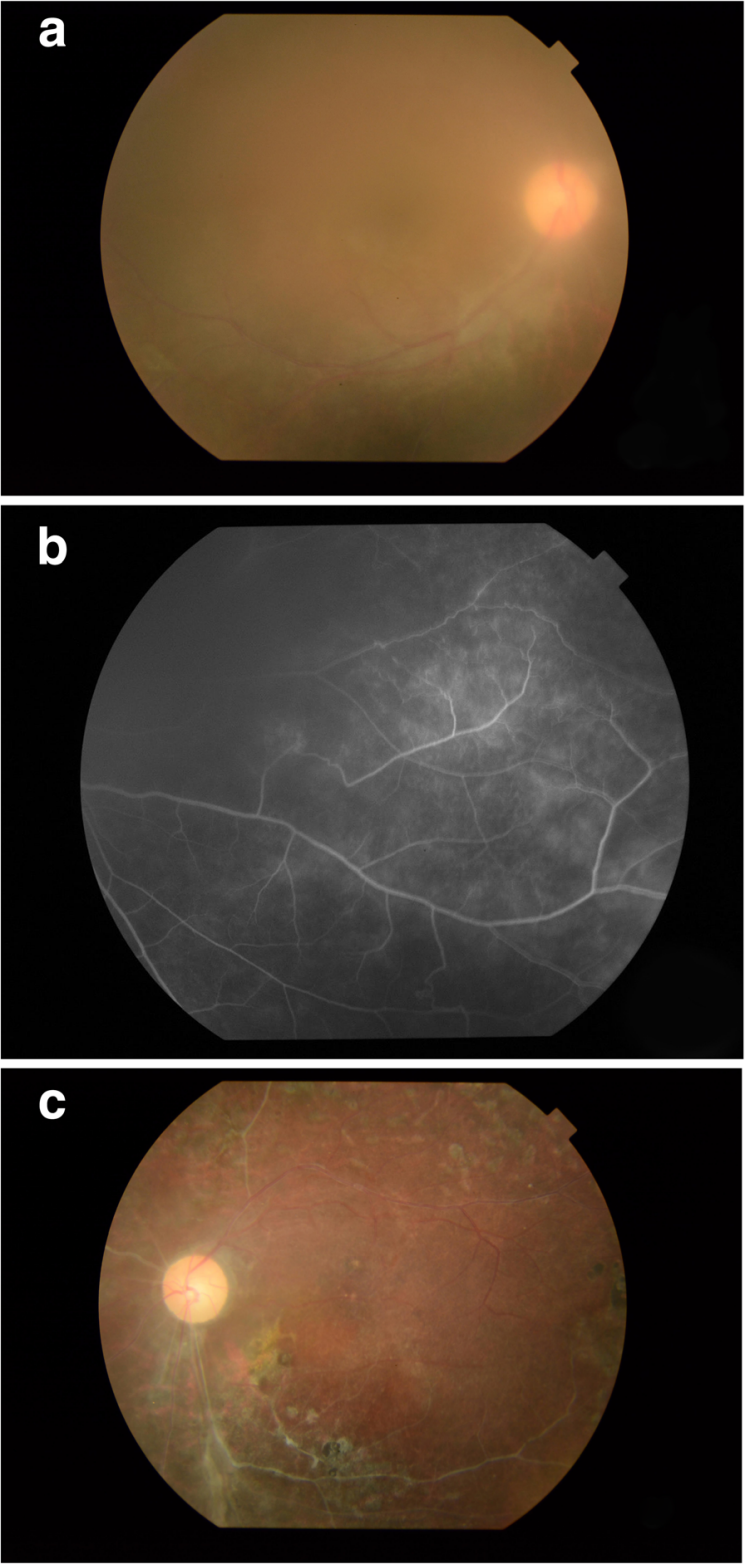
**a** Patient 2 presented with dense vitritis during ocular relapses before adalimumab use. **b** Evidence of peripheral periphlebitis, with obliterative pattern affecting both the arteries and veins in peripheral retina of the same patient during relapse. **c** Fundus photo of the same patient showing the consequence of chronic inflammation of patient 2. It shows evidence of sclerotic and occluded retinal vessels after repeated episodes of retinal vasculitis and vascular occlusions

**Fig. 2:**
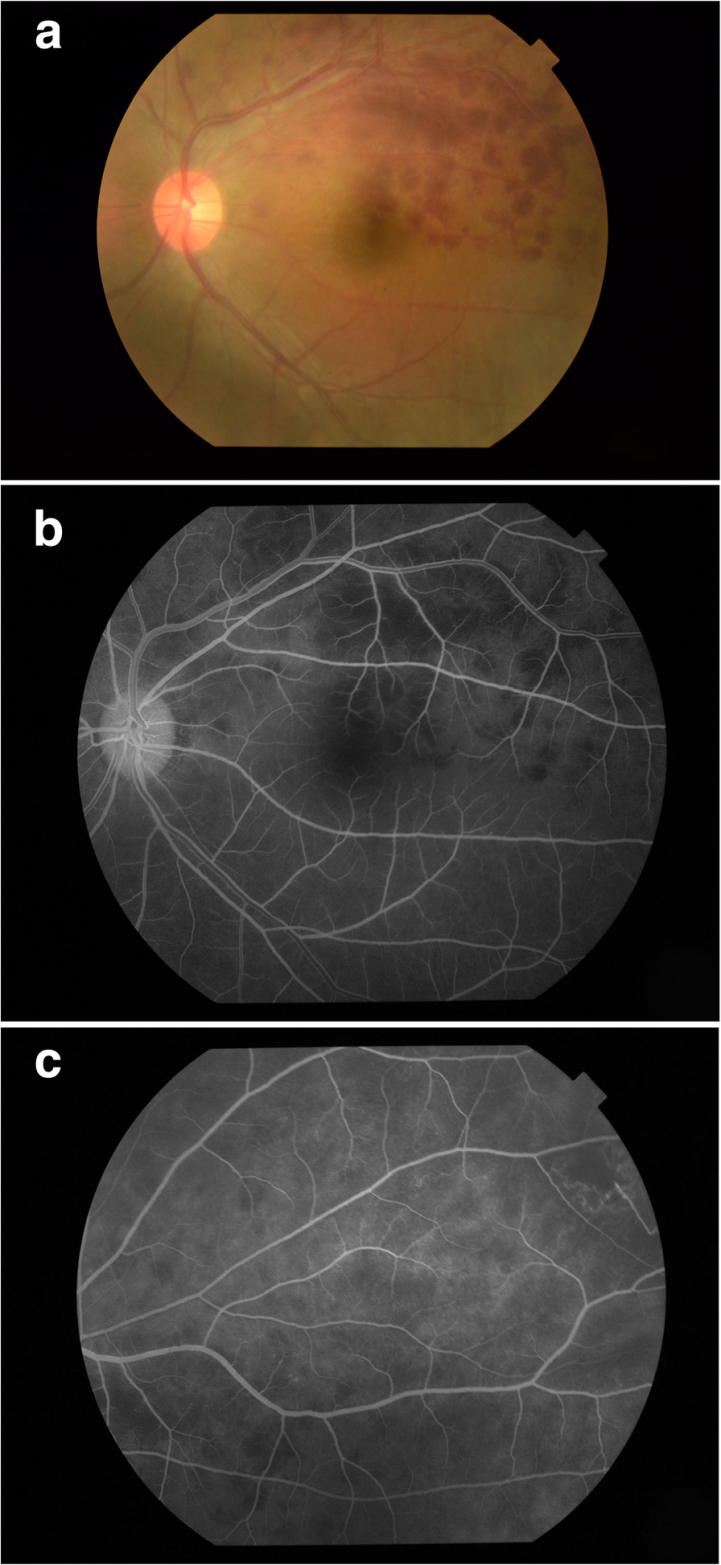
**a** Fundus photo from patient 3 showing evidence of obliterative, necrotising retinal vasculitis causing branch retinal artery occlusion with retinal haemorrhages during active ocular Behcet’s relapse. **b** Fluorescein angiography of the same patient showing evidence of vasculitis and non-perfused area. **c** shows an area of retinal ischaemia in the same patient with development of neovascularization of the retina

A total of 10 eyes of 5 patients were included. The mean age at disease onset was 14 (range, 9–18) years, and the mean ± SD disease duration of BD-related uveitis was 7.0 ± 5.0 years. All patients were male. The duration of adalimumab treatment ranged from 24 to 53 months. The clinical and demographic characteristics of these patients are presented in Table 1. The anatomic classification of uveitis was intermediate in one patient (2 eyes), and panuveitis in another 4 patients (8 eyes). Among the 8 eyes with panuveitis, 5 eyes presented with active retinal vasculitis. Before adalimumab treatment, one patient had bilateral secondary glaucoma and advanced visual field loss. Clinical presentations of patient 2 and 3 were presented in Figs. 1 and 2, respectively.

**Table 1:** Clinical and demographic characteristics of five children or adolescents affected with BD-uveitis

Patient no.	1	2 (Fig. 1)	3 (Fig. 2)	4	5
Sex	M	M	M	M	M
Ethnic origin	Chinese	Chinese	Chinese	Chinese	Chinese
Family history of BD	Nil	Yes	Nil	Nil	Nil
Age at uveitis onset	11	9	17	15	20
Disease duration (years)	7.5	15	5	6	1.5
Ophthalmic disease signs	Panuveitis Glaucoma	Bil. Retinitis Bil. Retinal vasculitis OS tractional retinal detachment OD sclerosed peripheral retinal vessels	Bil. Retinitis Bil. Retinal vasculitis OS BRAO OD NVE with sect PRP	Bil. Retinitis Bil. Retinal vasculitis OS tractional retinal detachment	Bil. Panuveitis Bil. Retinitis OD NVD
Rheumatic disease signs		Inflammatory bowel disease, complicated with perforated bowel Arthralgia Headache Erythema nodosum	Oral aphthae Arthralgia Abdominal pain	Oral aphthae Arthralgia Abdominal pain Pseudo-folliculitis with erythema nodosum	Oral aphthae Arthralgia Abdominal pain

*Bil.* bilateral, *BRAO* branch retinal artery occlusion, *BD* Behcet’s disease, *M* male, *NVD* neovascularization of the disc, *NVE* neovascularization elsewhere

All patients continued to receive immunosuppressive therapy and systemic corticosteroid at a lower dose after starting adalimumab therapy. Concomitant immunosuppressive therapies at baseline were systemic corticosteroids (*n* = 5), methotrexate (*n* = 1), cyclosporine A (*n* = 5) and azathioprine (*n* = 4). One patient (patient 3) was started with infliximab for 6 months before switching to adalimumab.

The results of the clinical response of individual patients are shown in Table 2. In 10 eyes, 8 eyes presented with features of panuveitis and 5 eyes with evidence of grade 2 retinal vasculitis involving the posterior pole. All eyes had complete resolution of inflammation, which was defined as having < 0.5+ cellular reaction in anterior chamber and vitreous and disappearance of signs of vasculitis on fundus examination and fluorescein angiography. Complete resolution of inflammation was noted after a mean of 3.4 weeks (median = 4 weeks; range = 2–4 weeks).

**Table 2:** Clinical response and treatment efficacy of adalimumab in BD-related uveitis

Patient no.	1	2	3	4	5
Before adalimumab (ADA) treatment					
Uveitis presentation	Bilateral intermediate uveitis	Bilateral panuveitis	Bilateral panuveitis	Bilateral posterior uveitis	Bilateral posterior uveitis
Retinal vasculitis	No	Yes	Yes	Yes	Yes
VA of OD, OS	OD 20/120, OS 20/120	OD 20/30, OS HM	OD 20/60, OS 20/40	OD 20/200, OS 16/200	OD 20/60, OS 20/40
No. of relapses per year	7	3	6	5	4
CS dosage prior to ADA treatment (mg)	10	20	70	40	60
CS-related complications	Cushingoid features Adrenal insufficiency Short stature	Short stature Osteoporosis	Nil	Cushingoid features	Cushingoid features
Concomitant DMARDs use at baseline	CSA 50 mg BD AZA 75 mg daily	CSA 50 mg BD AZA 75 mg daily	CSA 125 mg BD MTX 20 mg daily	CSA 50 mg BD AZA 75 mg daily	CSA 75 mg BD AZA 100 mg daily
Intolerability/side effects of DMARDs	Borderline HT	Intolerant to AZA due to abdominal pain	Nil	Borderline raised creatinine level	Nil
Ocular complication from BD-uveitis	Bilateral glaucoma Bilateral cataract	OS tractional retinal detachment	OS branch retinal artery occlusion	OS tractional retinal detachment	OD retinal neovascularization
Operation performed related to complication	Bilateral cataract operation OS trabeculectomy	OS vitrectomy and TRD repair	OS retinal laser therapy	OS vitrectomy and TRD repair	Nil
During adalimumab treatment					
Status of retinal vasculitis	NA	Subsided	Subsided	Subsided	Subsided
VA of OD, OS	OD 20/40, OS 20/40	OD 20/20, OS HM	OD 20/16, OS 20/16	OD 20/30, OS 8/200	OD 20/30, OS 20/16
Time to control of activity	2 weeks	4 weeks	3 weeks	2 weeks	2 weeks
No. of relapses in first year (0–12 months)	3	1	0	0	0 (0–6 months data only)
No. of relapses in second year (12–24 months)	3	0	3	2	NA
Nature of relapses	Anterior uveitis	Mild vitritis	Vitritis, focal retinitis, vasculitis causing BRAO	Vitritis	NA
CS dosage at 6 months after initiation of ADA	1 mg alt day (for adrenal insufficiency)	10 mg	9 mg	10 mg	7.5 mg
Change of concomitant DMARDs	Nil	Reduction in dosage of CSA and MMF	Nil	Halved dosage of CSA	Nil
Treatment for flare up	Topical steroid drops	Nil	High-dose oral steroid (1 mg/kg/day)	Controlled by increasing oral steroid	Nil
Remission	Yes	Yes	No	Yes in first 12 months	Yes
Long-term follow-up					
Adalimumab treatment duration	40 months	25 months	24 months	41 months	7 months
Adalimumab discontinuation	No	Yes (remission)	No	No	No
Relapse after adalimumab discontinuation	NA	No	NA	NA	NA
Length of follow-up from adalimumab initiation	40 months	36 months	38 months	41 months	7 months

*ADA* adalimumab, *CS* corticosteroid, *CSA* cyclosporine A, *MMF* mycophenolate mofentil, *DMARDs* disease-modifying antirheumatic drugs

During adalimumab treatment, four (80%) patients had complete control of ocular inflammation without any relapse in the 24-month period of follow-up. During the first 12 months of adalimumab treatment, one patient developed an episode of relapse and presented as dense vitritis. During the second year of adalimumab treatment, the same patient developed another episode of relapse with retinal vasculitis affecting the posterior pole with accompanying features of branch retinal artery occlusion (BRAO). Disease control with complete response was achieved after increasing the dosage of systemic corticosteroid and adjustment of immunosuppressant therapy (Fig. 2).

The mean dose of oral prednisolone was 40 mg (range, 10–70 mg) before initiation of adalimumab, and the mean dose was reduced to 7.7 mg (range, 1–11 mg) at 6 months after treatment. The mean logMAR ± SD BCVA was 0.712 ± 0.638 at baseline and improved to 0.172 ± 1.05 at 12 months (*P* < 0.001, paired sample *t* test)

Efficacy of adalimumab was maintained at long term. Beside from patient 3, all patients were able to achieve disease control during the follow-up period. Patient 1, 2 and 4 were able to remain relapse free for 50, 37 and 53 months, respectively. Systemic steroid was discontinued in patient 1 and 4, while patient 2 was on tapering dose of oral steroid down to 7 mg daily at latest follow-up.

Adalimumab was well tolerated; no adverse events of injection site reaction or increased infection rates were observed. All the patients continued to receive adalimumab injections until the last follow-up.

## Discussion

Paediatric uveitis represents up to 13.8% of patients in uveitis clinics and deserves special attention because of its therapeutic challenges [1, 12, 32]. The risk of poor visual outcome in paediatric age group is high. Clinicians face huge challenges while commencing steroids for paediatric group as extensive use of corticosteroids in children can have significant side effects. Biologics, especially anti-TNF alpha agents, are some of the viable options for treating sight-threatening uveitis.

It is recommended in the EULAR consensus that anti-TNF alpha could be considered as first line therapy in cases with severe sight-threatening BD-related uveitis [18]. In recent years, various studies have shown that adult patients suffering from sight-threatening BD-uveitis were successfully treated with anti-TNF alpha drugs [3, 19, 24–27, 35, 36]. The efficacy of adalimumab in non-infectious uveitis was reported in many prospective and retrospective studies in adult group. BD-related uveitis represents 6–33% of uveitis causes in these reports. The reported efficacy of adalimumab in these studies was 38–90% in controlling inflammation. Different outcome parameters were adopted in these studies, including complete disease quiescence, a two-step decrease in anterior chamber inflammation and a complete glucocorticoid sparing effect [8, 17, 20, 22, 34]. Among these studies, Diaz-Llopis et al. [8] reported satisfactory outcome in a relative large sample size of 131 cases (9.9% BD-related uveitis) in a relative young age group(mean age of 27 years old), revealing the possibility of higher prevalence of aggressive uveitis in younger patients.

FDA has recently extended the approval of adalimumab for the treatment of uveitis to include paediatric patients more than 2 years old. Promising results for anti-TNF alpha treatment in paediatric uveitis were available in case series [2, 5, 15]. In comparison to BD-related uveitis, more evidence was available for JIA uveitis. These reports were able to show a rapid response in control of inflammation, and a reduced rate of ocular complication by commencing anti-TNF alpha agents. The Sycamore trial has shown promising results with adalimumab therapy in JIA-associated uveitis [29]. Vazquez-Cobian and colleagues described a reduction in uveitis in 80.8% paediatric cases receiving adalimumab [41]. Biester et al. showed that the majority of patients (86%) with refractory uveitis were able to achieve 2 grade reduction in anterior chamber reaction with adalimumab use [5]. In comparison to JIA related uveitis, the evidence of adalimumab on paediatric BD-related uveitis is limited and data were scattered among reports that include various underlying causes of uveitis. Recently, Deitch et al. reported that 84.2% of cases could achieve complete steroid sparing effect by anti-TNF alpha therapy, which was based on a paediatric cohort consisting of 16.7% BD-related uveitis. However, BD-related uveitis in younger patients tends to present with more aggressive posterior uveitis [26]. Given the small sample size, our study aims to contribute more data on the effectiveness of adalimumab in treating paediatric Behcet’s uveitis.

In our study, we described a case series of several adolescents and young adults with BD who presented with aggressive panuveitis. Prompt remission of BD-related uveitis may be achieved with high-dose corticosteroids; however, most children developed steroid-related side effects and steroid dependence. In our study, two patients suffered from short stature, Cushingoid features and adrenal gland insufficiency after long-term steroid use. Despite aggressive treatment with a combination of immunosuppressive medications, active uveitis with retinitis and retinal vasculitis were observed in all our patients upon tapering of systemic steroid. In concordance with previous studies, blindness rate is high despite aggressive treatments in BD-related uveitis [37]. Our study describes the aggressive nature and high incidence of blinding in a limited number of paediatric BD-related uveitis.

There were no adverse events detected in our long-term follow-up of this case series related to adalimumab use. There was no reported incidence of allergic reactions, injection site reaction or demyelination, severe infection or tuberculosis infection related to adalimumab use. ANA level was checked at baseline and regularly after commencing adalimumab; no lupus-like syndrome was reported. The few cases in the current study tolerated well to adalimumab use in the long term.

The main limitations of our study included the small sample size and lack of control comparison. Despite this, this study demonstrated good clinical response to adalimumab in a small series of severe, aggressive, sight-threatening BD patients presenting at a young age. Our series had a long follow-up period of up to 4 years with a median follow-up duration of 40 months. In the long term, efficacy of adalimumab may change due to development of antidrug antibodies [4]. This phenomenon is not observed in our series yet by using concomitant anti-metabolites.

In conclusion, we described the clinical response of a case series of patients with BD at adolescent onset being treated with adalimumab. Overall, all patients with ocular involvement showed complete resolution of inflammatory ocular involvement with fewer episodes of ocular relapse. These patients were able to maintain their baseline BCVA at last visit. Our study showed adalimumab has great potential for treating paediatric BD-related uveitis but further large-scale studies are warranted.

## Data Availability

Data was available and summarized in a table, which was presented in the manuscript.

## Abbreviations

BD: Behcet’s disease
BRAO: Branch retinal artery occlusion
JIA: Juvenile idiopathic arthritis
SUN: Standardization of Uveitis Nomenclature
TNF: Anti-tumour-necrotic factor
